# Integration of deep learning with Ramachandran plot molecular dynamics simulation for genetic variant classification

**DOI:** 10.1016/j.isci.2023.106122

**Published:** 2023-02-02

**Authors:** Benjamin Tam, Zixin Qin, Bojin Zhao, San Ming Wang, Chon Lok Lei

**Affiliations:** 1 Ministry of Education Frontiers Science Center for Precision Oncology, Faculty of Health Sciences, University of Macau, Macau SAR, China; 2 Cancer Centre, Faculty of Health Sciences, University of Macau, Macau SAR, China; 3 Institute of Translational Medicine, Faculty of Health Sciences, University of Macau, Macau SAR, China

**Keywords:** Biological sciences, Genetics, Systems biology

## Abstract

Functional classification of genetic variants is a key for their clinical applications in patient care. However, abundant variant data generated by the next-generation DNA sequencing technologies limit the use of experimental methods for their classification. Here, we developed a protein structure and deep learning (DL)-based system for genetic variant classification, DL-RP-MDS, which comprises two principles: 1) Extracting protein structural and thermodynamics information using the Ramachandran plot-molecular dynamics simulation (RP-MDS) method, 2) combining those data with an unsupervised learning model of auto-encoder and a neural network classifier to identify the statistical significance patterns of the structural changes. We observed that DL-RP-MDS provided higher specificity than over 20 widely used in silico methods in classifying the variants of three DNA damage repair genes: *TP53*, *MLH1*, and *MSH2*. DL-RP-MDS offers a powerful platform for high-throughput genetic variant classification. The software and online application are available at https://genemutation.fhs.um.edu.mo/DL-RP-MDS/.

## Highlights

• Classifying genetic variants in ClinVar database by using RP-MDS and deep learning
• DL-RP-MDS achieved the highest specificity compared to over 20 in silico methods
• Demonstrated with variant classification for DNA damage repair genes: *TP53*, *MLH1*, and *MSH2*
• Online platform available at https://genemutation.fhs.um.edu.mo/DL-RP-MDS/

## Graphical abstract

**Figure:**
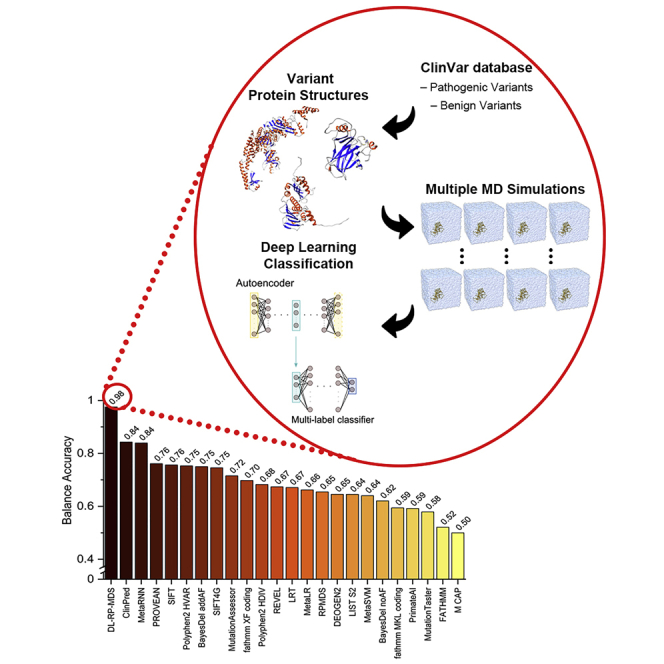

## Introduction

Next-generation DNA sequencing technologies allow the collection of a massive quantity of genetic variation data at the population level, with the majority as single base variants. Although identifying the genetic variants causing apparent damage in protein structure can be straightforward, determining the functional impact of missense variants, in which a single base variant causes an amino acid change in a protein, is challenging as they mainly affect local rather than global protein structure. Currently, a large quantity of missense variants identified in the human genome remains unclassified.^1^^,^^2^ For example, of the 56,483 missense variants identified in 170 DNA damage repair (DDR) genes, 50,427 (89.3%) remain as variant of uncertain significance (VUS).^3^ The lack of functional information for the genetic variants limits their clinical applications.^4^ Although many in silico tools have been developed with the aim of determining the functional impact of missense variants, the American College of Medical Genetics and Genomics and the Association for Molecular Pathology (ACMG-AMP) guidelines conclude that the accuracy of these methods remains in question.^5^

From the atomistic point of view, the functionality of a protein is determined by its structure maintained by intramolecular and intermolecular interactions through electrostatic, hydrogen bonding, Van der Waal, etc. As such, the impact of a genetic variant on protein function can be reflected by its impact on protein structural stability. In our previous study, we developed the Ramachandran plot-molecular dynamics simulation (RP-MDS) method to measure the impact of missense variants on protein structure. In the process, the torsion angle phi (φ) and psi (Ψ) of the protein secondary structural backbone are assimilated throughout MD trajectories. The alteration of backbone information reflects the impacts of the altered residue on protein structure.^6^ Applying RP-MDS, we were able to classify multiple TP53 VUS.^6^^,^^7^ However, there are several limitations in RP-MDS, including the need to manually define the cut-off value in order to separate between deleterious and non-deleterious variants, the difficulty to analyze the genes with insufficient known benign and pathogenic variants as the training data, and the challenge to measure minor structural changes often masked within the statistically averaged values.

**Figure 1:**
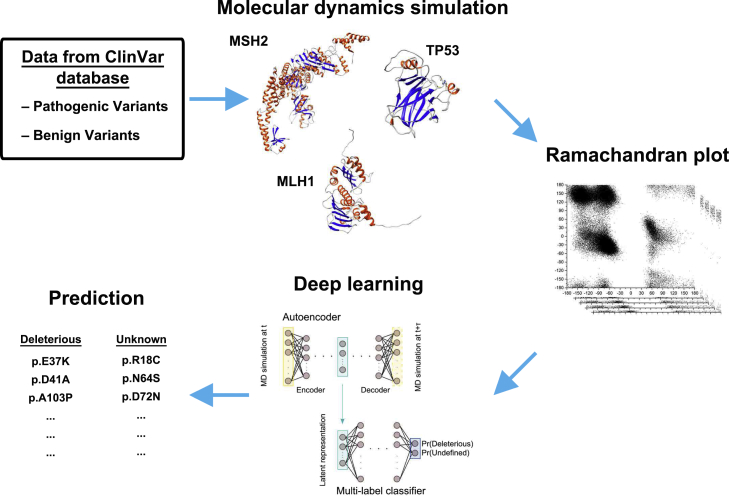
Procedures of DL-RP-MDS Missense variants in TP53, MLH1, and MSH2 were extracted from ClinVar, and used as training and testing datasets. MDS generated trajectories of the corresponding protein structure. Benign and pathogenic variant RSPs were extracted from the trajectories and used as the input of the DL pipeline. Autoencoder and neural network classifier identified the energy landscape of the RSP, predicted the deleteriousness of the missense variant, and categorized it as either “deleterious” or “unknown”.

Deep learning (DL) is increasingly applied in molecular biology studies.^8^^,^^9^^,^^10^ We hypothesized that the integration of DL with RP-MDS may significantly increase the power of RP-MDS for genetic variant classification. To test our hypothesis, we included the two approaches to form the DL-RP-MDS method. DL-RP-MDS combined an unsupervised learning model, the auto-encoder (AE), with a multi-layer neural network classifier to generate a probabilistic classification.^11^^,^^12^ AE is a special class of neural network. It can match its outputs to its inputs through model learning and compress the high-dimensional input space to a low-dimensional latent space (encoder). The encoder then can prioritize the input with the high information density and the complex relationship between the inputs. The decoder (the second part of AE) then reconstructs an output with the same dimensions as the inputs from the latent space, with the dataset adhering to the rules learned from the encoder. AEs share the same concept as other dimension-reduction models, such as principal components analysis (PCA) and multidimensional scaling (MS). However, AEs are more suitable for highly nonlinear data, such as those generated by RP-MDS.^13^^,^^14^ Furthermore, the Synthetic Minority Oversampling TEchnique (SMOTE) in DL was used to address the issue of imbalanced training data, i.e., the known benign and deleterious variants.^15^ Imbalanced data are well known to pose issues in classification,^16^^,^^17^ as these imbalanced training data may skew the classification toward (majority) deleterious variants by ignoring the (minority) benign region. SMOTE enhances recognition of the minorities dataset by broadening and strengthening the region by generating “synthetic” training data. In the process, the minority feature regions are created by joining any or all the minority from the nearest neighbors. By inserting random samples within the minority region, SMOTE effectively turns the decision region toward more general to enhance the regional contrast between benign and deleterious variants to facilitate variant classification. An overview of the DL-RP-MDS approach is shown in Figure 1.

By testing the missense variants from the human tumor suppressor gene *TP53*,^18^ and DNA mismatch repairs *MLH1*, and *MSH2*,^19^^,^^20^ we show that DL-RP-MDS can successfully classify missense variants with over 98% balanced accuracy (BA), demonstrating that DL-RP-MDS is better than most of the widely used computational methods for genetic variant classification. Overall, the study provides a road map for the application of DL to assess missense variants.

## Results

### Construction of mutant protein structures

A total of 81 pathogenic and 24 benign/likely benign *TP53* variants, 45 pathogenic and 8 benign *MLH1* variants, and 38 pathogenic and 12 benign/likely benign *MSH2* missense variants were selected from the ClinVar database (Table S1). TP53 crystal structure (PDB ID: 2OCJ, resolution 2.05 Å, composed of DNA binding domain 94–313 residues), MLH1 crystal structure composed of ATPase domain (1–207) and MutS homologs interaction domain (208–346) (PDB ID: 4P7A, resolution 2.30 Å), and MSH2 crystal structure composed of the whole MSH2 protein structure (PDB ID: 3THX, resolution 2.7 Å) were used as the templates to build the mutant structure for each variant.

There were, in total, 44 benign/likely benign variants for *TP53*, *MLH1*, and *MSH2*; 35 variants had a “three-star review status” (criteria provided, multiple submitters, reviewed by the expert panel), four variants had a “one-star review status” (criteria provided, single submitter), and five variants changed their classifications to variants of uncertain significance (VUS) or conflicted interpretation (Tables S1A–S1C).^3^ For all the 164 pathogenic variants, 85 variants had the “three-star review status”, 51 variants had a “two-star review status” (criteria provided, multiple submitters), and 28 variants had the "one-star review status". Functional data provided by International Agency for Research on Cancer (IARC), Leiden Open Variation Database (LOVD), and UniProt showed that most of the variants had a strong positive correlation to the pathogenicity classification.^21^^,^^22^^,^^23^

### RP-MDS for classifying missense variants

**Figure 2:**
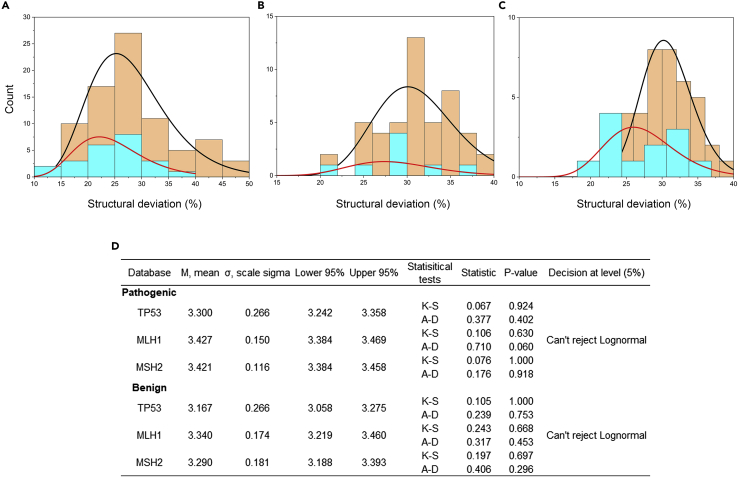
Goodness of fit test for the missense variants in TP53, MLH1, and MSH2 It showed the distribution of structural deviation for pathogenic and benign variants. (A) TP53; (B) MLH1; (C) MSH2; (D) Summary of the statistical tests. K-S: Kolmogorov-Smirnov; A-D: Anderson-Darling; Peach: pathogenic variants; cyan: benign variants; red line: lognormal distribution curve for benign variants; black line: lognormal distribution curve for pathogenic variants.

We first used the data generated from the known benign and pathogenic variants to determine the cut-off values between the deleterious and non-deleterious variants. Lognormal distribution was fitted against the benign and pathogenic distribution (Figure 2). Kolmogorov-Smirnov and Anderson-Darling goodness-of-fit test accepted lognormal distributions for all genes (Figure 2D).^24^^,^^25^ RP-MDS was able to classify benign and pathogenic variants in each gene as the benign variant distribution curves differed significantly from the distribution of the pathogenic variants. We determined the optimal cut-off points as 3.17 (true negative, TN = 58.3%; true positive, TP = 58.0%) for TP53, 3.38 (TN = 75.0%, TP = 71.1%) for MLH1, and 3.36 (TN = 66.7%, TP = 65.8%) for MSH2. The results for individual variants based on the settled cut-off positions were obtained (Tables S2 and S3).

### DL-RP-MDS for classifying missense variants

**Figure 3:**
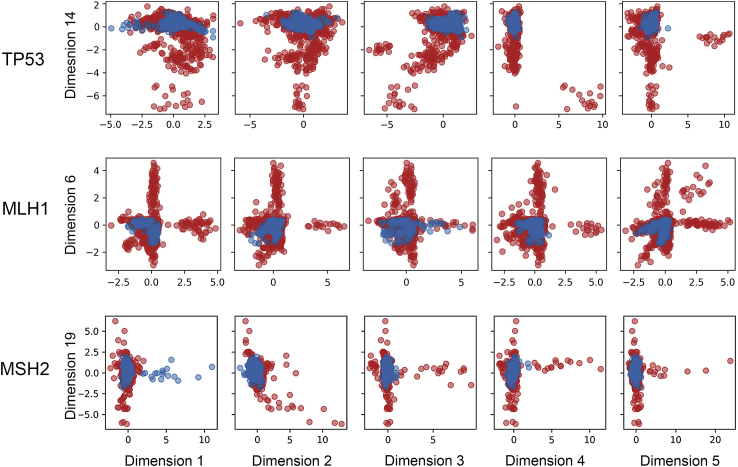
Examples of the latent dimensions generated by the autoencoder DL-RP-MDS reduced the complexity of Ramachandran scatterplots but retained the crucial information. The combination of the unique information in each dimension was used as the classification criteria. Blue: Benign variants; Red: Pathogenic variants.

The Ramachandran scatter plots (RSP) of benign and pathogenic variants generated by molecular dynamics simulation (MDS) were directed into AE (see Figure 1). The optimized hyperparameter configuration for the classifier was a fully connected neural network with one hidden layer of 1024 neurons and without dropout, together with a latent representation dimension of *q* = 14 for TP53, *q* = 8 for MLH1 and *q* = 20 for MSH2. The values were chosen based on the validation accuracy ≈95%. These various latent representation dimensions were employed to characterize the relationship between known benign and pathogenic variants (Figures S1–S3). Examples of graphical illustrations for the latent dimensions were shown in Figure 3. Benign variants occupied a localized region and partially overlapped with pathogenic variants, which represented structural features observed in the benign and pathogenic variants. In contrast, the unique regions occupied by the pathogenic variants were interpreted as localized distinct protein deformities caused by the missense variants. For each variant, probabilities of “deleterious, D” and “unknown, U” were assigned (Table S2). Variants used as part of the training data were tested by the method again and scored with high probability (>90%) in their respective classifications. Incorrectly identified variants (false negative, FN; false positive, FP) by DL-RP-MDS with probabilities close to ∼50% implied that the protein structure was at the threshold of benign/pathogenic transitions.

### Stratified cross-validation results

**Figure 4:**
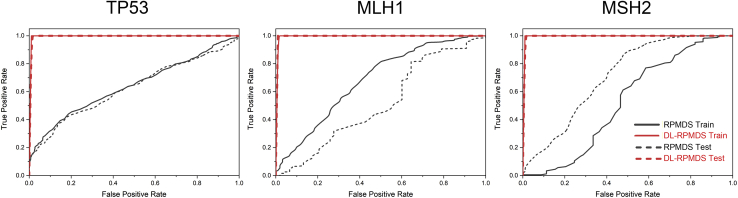
Receiver operating characteristic (ROC) curves for DL-RP-MDS and RP-MDS Each ROC curve was the average of 20 stratified cross-validation models. For DL-RP-MDS, variants were grouped by frames. The overlapped testing dataset and training dataset ROC illustrated the equal area under the curve (AUC) for TP53 (left), MLH1 (center), and MSH2 (right). For RP-MDS, the AUC of the testing and training datasets was comparable for TP53, lower for MLH1, and higher for MSH2. Red solid line: DL-RP-MDS training dataset model; Red dotted line: DL-RP-MDS testing dataset model; Black solid line: RP-MDS training dataset model; Black dotted line: RP-MDS testing dataset model.

RP-MDS underwent a four-fold stratified cross-validation test with five repeats using the TP53, MLH1, and MSH2 variants, and their receiver operating characteristic (ROC) curves were computed. Each model was fitted against the lognormal distribution and was accepted by the Kolmogorov-Smirnov (K-S) and Anderson-Darling (A-D) goodness-of-fit test (not shown). The average area under the ROC curve (AUC) values for the training and testing datasets were 0.64 and 0.62 for TP53, 0.69 and 0.50 for MLH1, 0.72 and 0.54 for MSH2, respectively (Table 1, Figure 4). Although the training datasets performed moderately well across the three genes, the limited number of benign variants in MLH1 and MSH2 caused a significant reduction of accuracy in the testing datasets, as the RP-MDS training data were dependent on the structural deviation range of benign variants.

**Table 1:** The average of four-fold stratified sampling with five repeats

Gene	AUC		
	RP-MDS	DL-RP-MDS	
		Variants	Frames
**Training**			

TP53	0.64	1.00	1.00
MLH1	0.69	1.00	1.00
MSH2	0.72	0.98	1.00

**Testing**			

TP53	0.62	0.74	1.00
MLH1	0.50	0.57	1.00
MSH2	0.54	0.54	1.00

DL-RP-MDS also underwent the same four-fold stratified cross-validation test with five repeats. Here, individual frames of RSP were used for the training and testing datasets, and two different grouping strategies were utilized. One was grouped by variants, for which DL-RP-MDS treated the 334 RSP for each variant as one sample; the other was grouped by frames, for which DL-RP-MDS treated the 334 RSP for each variant as different individual samples. Each grouping strategy undertook randomized permutation before the stratified sampling. The strategy of “grouped by variants” showed that the average AUC values for the training and testing datasets were 1.00 and 0.74 for TP53, 1.00 and 0.57 for MLH1, 0.98 and 0.54 for MSH2, respectively (Table 1 and Figure S4). Although the training dataset outperformed RP-MDS, the testing dataset was only marginally better than RP-MDS. This grouping method was used to have an objective comparison with RP-MDS; thus, the model appeared to be overfitted. Although the lack of benign variants contributed to the low testing score for DL-RP-MDS, significant improvements in the testing data were achieved when using the strategy of “grouped by frames” (Figure 4). This grouping method provided a better description of DL-RP-MDS operation, as it treated each RSP as an individual. DL-RP-MDS achieved 1.00 and 1.00 for the testing and training data across TP53, MLH1 and MSH2. The results of the four-fold stratified cross-validation demonstrated that DL-RP-MDS models were not overfitted and performed better than RP-MDS.

### Comparing RP-MDS and DL-RP-MDS with 22 in silico methods

Multiple in silico computational methods have been developed based on different principles, such as familial segregation,^26^ evolution conservation,^27^ classical statistics,^28^ experiment assays,^29^ and combination of different principles.^30^^,^^31^^,^^32^^,^^33^^,^^34^^,^^35^^,^^36^^,^^37^^,^^38^^,^^39^^,^^40^ Missense variants in TP53, MLH1, and MSH2 were used to compare the performance of DL-RP-MDS and RP-MDS against 22 commonly used in silico methods: SIFT, SIFT4G, PolyPhen2_HDIV, PolyPhen2_HVAR, LRT, MutationTaster, MutationAssessor, FATHMM, PROVEAN, MetaSVM, MetaLR, MetaRNN, M_CAP, REVEL, PrimateAI, DEOGEN2, BayesDel_AddAF, BayesDel_noAF, ClinPred, LIST_S2, FATHMM_MKL_coding, and FATHMM_XF_Coding through utilizing dbNSFP (Tables 2 and S3).^41^ We quantified each method by determining the sensitivity, which was calculated by the number of TP predictions divided by all the number of pathogenic samples, and the specificity, which was calculated by the number of TN predictions divided by all the number of benign samples. For the pathogenic variants, 21 in silico methods except PrimateAI had the sensitivity between 78 and 100%; BayesDel_addAF, FATHMM, M_CAP, REVEL, and BayesDel_addAF reached 100%, and PrimateAI had 25%, the lowest score among all 22 in silico methods. In comparison, DL-RP-MDS reached 95% sensitivity; for the benign variants, only two (PrimateAI, PolyPhen2_HVAR) out of the 22 in silico methods had specificity >70%, in which PrimateAI reached 93.2%, the highest among all 22 in silico methods specificity, the remaining 20 in silico methods showed specificity in the range of 0–70%. In comparison, DL-RP-MDS achieved 100% specificity, the highest over all the in silico methods.

**Table 2:** Comparison of DL-RP-MDS with 22 in silico methods

Methods	TP53			MLH1			MSH2			Average		
	Sen∗	Spec^†^	BA^‡^	Sen∗	Spec^†^	BA^‡^	Sen∗	Spec^†^	BA^‡^	Sen∗	Spec^†^	BA^‡^
DL-RP-MDS	0.85	1.00	0.93	1.00	1.00	1.00	1.00	1.00	1.00	0.95	1.00	0.98
ClinPred	1.00	0.75	0.88	1.00	0.75	0.88	0.97	0.58	0.78	0.99	0.69	0.84
MetaRNN	1.00	0.71	0.85	0.98	0.63	0.80	0.97	0.75	0.86	0.98	0.69	0.84
PROVEAN	0.96	0.88	0.92	0.93	0.38	0.65	0.92	0.50	0.71	0.94	0.58	0.76
SIFT	1.00	0.71	0.85	0.93	0.50	0.72	0.89	0.50	0.70	0.94	0.57	0.76
PolyPhen2_HVAR	0.88	0.88	0.88	0.71	0.63	0.67	0.76	0.67	0.71	0.78	0.72	0.75
BayesDel_addAF	1.00	0.54	0.77	1.00	0.38	0.69	1.00	0.58	0.79	1.00	0.50	0.75
SIFT4G	0.96	0.75	0.86	0.87	0.50	0.68	0.89	0.50	0.70	0.91	0.58	0.75
MutationAssessor	0.99	0.83	0.91	0.89	0.25	0.57	1.00	0.33	0.67	0.96	0.47	0.72
fathmm_XF_coding	0.96	0.75	0.86	0.96	0.13	0.54	0.97	0.42	0.70	0.96	0.43	0.70
PolyPhen2_HDIV	0.88	0.83	0.85	0.84	0.25	0.55	0.79	0.50	0.64	0.84	0.53	0.68
REVEL	1.00	0.54	0.77	1.00	0.25	0.63	1.00	0.25	0.63	1.00	0.35	0.67
LRT	0.94	0.75	0.84	1.00	0.25	0.63	0.92	0.17	0.54	0.95	0.39	0.67
MetaLR	1.00	0.00	0.50	0.89	0.50	0.69	1.00	0.58	0.79	0.96	0.36	0.66
RP-MDS	0.58	0.58	0.58	0.69	0.75	0.72	0.66	0.67	0.66	0.64	0.67	0.65
DEOGEN2	1.00	0.17	0.58	0.96	0.25	0.60	1.00	0.50	0.75	0.99	0.31	0.65
LIST_S2	0.88	0.63	0.75	0.98	0.00	0.49	0.97	0.42	0.70	0.94	0.35	0.64
MetaSVM	1.00	0.00	0.50	0.87	0.50	0.68	0.97	0.50	0.74	0.95	0.33	0.64
BayesDel_noAF	1.00	0.42	0.71	1.00	0.25	0.63	0.97	0.08	0.53	0.99	0.25	0.62
fathmm_MKL_coding	0.96	0.54	0.75	0.98	0.00	0.49	1.00	0.08	0.54	0.98	0.21	0.59
PrimateAI	0.12	1.00	0.56	0.27	0.88	0.57	0.37	0.92	0.64	0.25	0.93	0.59
MutationTaster	0.83	0.54	0.68	0.93	0.00	0.47	0.92	0.25	0.59	0.89	0.26	0.58
FATHMM	1.00	0.00	0.50	1.00	0.13	0.56	1.00	0.00	0.50	1.00	0.04	0.52
M_CAP	1.00	0.00	0.50	1.00	0.00	0.50	1.00	0.00	0.50	1.00	0.00	0.50

∗Sen = Sensitivity; ^†^Spec = Specificity; ^‡^BA = Balance Accuracy.

**Figure 5:**
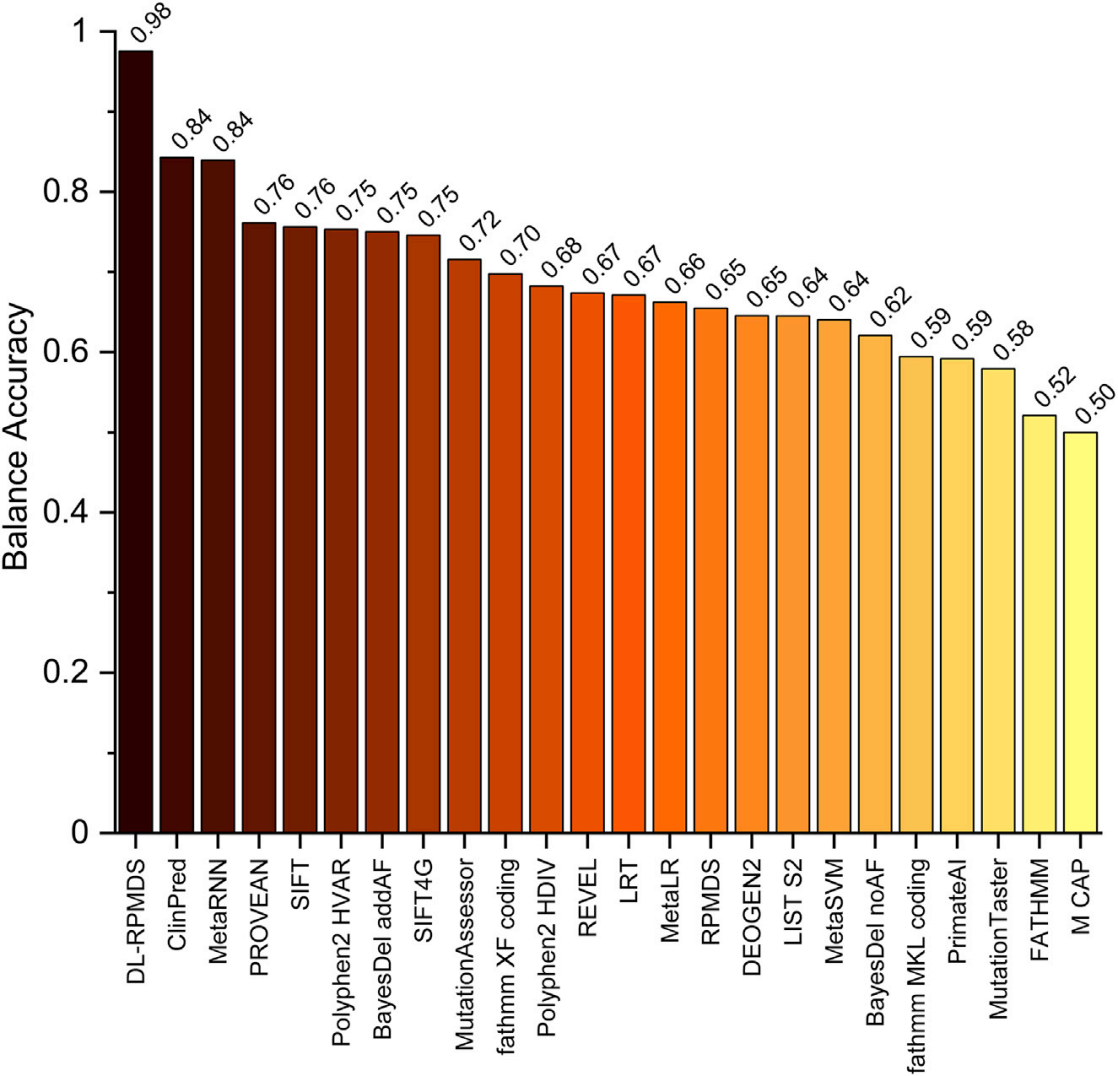
Comparison of balance accuracy of DL-RP-MDS, RP-MDS, and 22 in silico methods DL-RP-MDS scored the highest among all methods. The maroon to yellow colors represents the scores from high to low.

The overall performance was summarized in Table 2. Most of the widely used in silico methods showed high sensitivity but at the cost of a low specificity resulting in high false-positive rates. DL-RP-MDS scored the highest among all the in silico methods tested, with a sensitivity of 0.95 and specificity of 1.00. For example, the commonly used in silico methods of SIFT, PolyPhen2, and MutationTaster achieved >0.73 in sensitivity but <0.42 in specificity, resulting in BAs of 0.65, 0.56, and 0.48, respectively (Figure 5). DL-RP-MDS obtained balance accuracies of 0.98, overperformed the 0.84 by ClinPred and Meta RNN, and 0.76 by PROVEAN. The results showed that DL-RP-MDS outperformed most of the existing in silico methods, provided that enough benign variants were available as the training data.

## Discussion

Computational approaches are promising for genetic variant classification as their high-throughput capacity allows the characterization of massive genetic variation data. Our approach for missense variant classification is based on the impact of the variants on protein structure stability. Our current study integrated RP-MDS and DL-RP-MDS into a single system for missense variant classification. In RP-MDS, MDS characterizes molecular conformation by allowing time-dependent evolution based on atomistic interactions. The information of φ and Ψ torsional angle from MDS trajectories is extracted to provide an energy landscape.^42^^,^^43^^,^^44^ Measurement of φ and Ψ torsional angle allows distinguishing energy landscapes between benign and pathogenic variants.

In contrast to the traditional view of the RSP, where the emphasis was on the residues in the disallowed region, RP-MDS directly measures the dynamical structure changes by RSP and determines the deleteriousness of the variants based on the average structure dynamics reflected in the last 10 ns of MDS. Deleterious variants were thought to have deleterious effects on the surrounding environment such that the averaging dynamics of the protein (i.e., converting RSP into Ramachandran density plot, RDP) may reveal the effects of the variant residue in the proteins. Thus, the variants with significant differences can be identified as “deleterious”. However, RDP can dampen the scrupulous details of structural change but mainly conserve significant information. DL-RP-MDS used RSP as input to conserve the vast information of the residue and backbones. It provides a high capability to distinguish energy landscapes between deleterious and benign variants. The first part of the DL approach, AE, nonlinearly compresses the individual RSP frame into the latent space;^45^ and the second part, the neural network classifier, performs the classification based on the latent space to provide a probability for benign and deleterious variants. Another benefit of DL-RP-MDS is that it provides a continuous value (between 0 and 1) for high-confident variant classification instead of an arbitrarily defined cut-off for binary classification. DL-RP-MDS substantially improves the accuracy of RP-MDS as reflected by its differentiation between benign and pathogenic variants at AUC of nearly 100% when each gene was evaluated individually. DL-RP-MDS substantially overcomes the problem of overprediction of deleterious by the current in silico methods.^46^^,^^47^

*TP53*, *MLH1*, and *MSH2* are distinct cancer genes^48^ and were used as examples to test DL-RP-MDS. TP53 is known as the “guardian of the genome”. Mutations in its DNA binding domain can affect the expression of a large number of genes causing undesirable functional change;^49^ MLH1 plays important roles in safeguarding the integrity of genomic DNA through its N-terminal ATPase domain and its MutS homolog interaction domain;^50^ MSH2 forms a heterodimer with MSH6 to make the human MutSα mismatch repair complex involved in transcription repair, homologous recombination, and base excision repair.^51^ The results from our study of the missense variants of these three genes demonstrate that the classification of missense variants by DL-RP-MDS needs to be gene-specific. This is different from the principles used by the 22 in silico methods tested in this study as they assume that the same principles apply to all genes. We consider that such an assumption ignores the structural differences between different genes, contributing to their lower accuracy. For example, SIFT and PolyPhen-2 use evolution conservation for variant classification. However, they are not be suitable to classify the missense variants in *BRCA1* and *BRCA2*, as the pathogenic variants in *BRCA1* and *BRCA2* were mostly originated in recent human history rather than evolution conservation from other species.^52^ On the contrary, DL-RP-MDS uses protein structure as the reference to identify abnormalities caused by missense variants by following the thermodynamics changes to differentiate benign and deleterious variants. Furthermore, DL-RP-MDS can tolerate the altered residues and allocate deleterious probability with high BA values. Our study indicates that structural change is a valuable property for variant classification.

In summary, DL-RP-MDS provides a highly accurate tool for missense variant classification. It is readily applicable to classify the missense variants of unknown significance and unclassified missense variants.

### Limitations of the study

Several limitations of DL-RP-MDS suggest future studies to advance the prediction of missense variants: 1) The limited number of benign variants may skew the variant test toward more deleterious identification, even in the presence of SMOTE. 2) Not all deleterious variants change protein structure. Therefore, DL-RP-MDS may not be able to characterize these variants. 3) Interpretation of DL results can be elusive because of the “black box” issue discussed in ref.^53^, although DL-RP-MDS avoids “black box” interpretation as phi-psi angle based on protein backbone. 4) DL parameters are subject to fine-tuning. Although layers and dimensions were tuned specifically for TP53, MLH1, and MSH2, these parameters can be protein specific. The accuracy of the prediction may be subject to variants classified by different resources (i.e., different databases) under distinct classification criteria. 5) DL-RP-MDS uses MDS to observe structural changes within the protein, which does not account for external molecules and therefore does not provide information or properties such as LOF or gain of function. Furthermore, only fragments of the protein domains were experimentally purified and crystallized, making the MD simulations and the analyses limited to the structures available in the PDB database.^54^

## STAR★Methods

### Key resources table

**Table:** 

REAGENT or RESOURCE	SOURCE	IDENTIFIER
**Software and algorithms**		

Python/3.2.7	Python	https://www.python.org/
numpy/1.19.5	Harris et al.,^55^	https://numpy.org/
scipy/1.6.0	Virtanen et al.,^56^	https://www.scipy.org/
Pandas/1.2.1	Pandas	https://pandas.pydata.org/
Joblib/1.0.0	Joblib	https://joblib.readthedocs.io/en/latest/
Sckit-learn/1.0.0	Pedregosa et al.,^57^	https://scikit-learn.org/stable/
Tensorflow/2.4.0	Dillon et al.,^58^	https://www.tensorflow.org/
Tensoflow-addons/0/13/0	Dillon et al.,^58^	https://www.tensorflow.org/addons/overview
Keras-tuner/1.0.2	Keras tuner	https://keras.io/keras_tuner/
Imbalanced-learn/0/.8.1	Lemaître et al.,^59^	https://imbalanced-learn.org/stable/
Seaborn/0.11.1	Waskom^60^	https://seaborn.pydata.org/
**DL-RP-MDS** online platform		https://genemutation.fhs.um.edu.mo/DL-RP-MDS/
DL-RP-MDS code		https://doi.org/10.5281/zenodo.7435215

### Resource availability

#### Lead contact

Further information and requests for resources and reagents should be directed to and will be fulfilled by the lead contact: Chon Lok Lei (chonloklei@um.edu.mo).

#### Material availability

This study did not generate new unique reagents.

### Experimental model and subject details

This study did not use any experimental model.

### Method details

#### Sources of missense benign and pathogenic variants

From the ClinVar database, we identified 81 known pathogenic and 24 known benign/likely benign missense variants located within the TP53 DNA binding domain, 45 known pathogenic and 8 known benign missense variants within the MLH1 N-terminus, 38 pathogenic and 12 benign missense variants in MSH2 whole structure (Table S1).^3^

#### MDS

TP53 DNA binding domain structure (PDB ID: 2OCJ, 94–313 residues, 2.05 Å resolution),^61^ MLH1 N-terminus (PDB ID: 4P7A, 0–348 residues, 2.30 Å resolution)),^50^ and MSH2 structure (PDB ID: 3THX, 1–934 residues, 2.70 Å resolution)^62^ were used as the templates to construct the mutant structures for each of the 207 missense variants following the procedure described in ref.^6^. Briefly, the software MODELLER in the Chimera package was used to build the missing atoms, and the Rotamer in the Chimera package was used to replace the template amino acid residue with the residue coded by the missense variant from the Dunbrack rotamer library.^63^^,^^64^ The mutant structure was used as the starting configuration for MD simulations for all missense variants using GROMACS (version 2020).^65^ AMBER14 force field was chosen to model the protein complex and the Mg ions.^66^ The protein structure was situated in a 10 × 10 × 10 nm simulation box for MLH1 and TP53, whereas MSH2 was situated in a box with the boundary 1 nm away from the protein. Each system was solvated with TIP3P water and neutralized with NA^+^ or Cl^−^ ions. Including the protein, TIP3P water, NA^+^, and Cl^−^ ions, each simulation system holds approximately ∼99,000 atoms for MLH1 and TP53, and ∼200,000 atoms for MSH2. Steepest descent algorithm was applied to the system before 1 ns equilibration run at 298 K and 1 bar in the NPT ensemble using Berendsen thermostat and barostat. The system was set at 298 K and 1 bar in the NPT ensemble by using a V-rescale thermostat and Parrinello-Rahman barostat during the 40 ns production run.^67^ The Verlet velocity algorithm was employed with a time step of 2 fs. Particle Mesh Ewald (PME) method was used to treat the long-range electrostatic interactions with the cut-off distance set at 1.0 nm. Hydrogen bonds were constrained at equilibrium lengths by using the LINC algorithm. The trajectory frame of MD was saved every 30 ps.^68^

#### Ramachandran plot

RSP for each variant was generated from the MD simulation trajectory following the procedure.^6^ The last 10 ns of the production run were used for RP-MDS and DL-RP-MDS analyses. For RP-MDS, the torsional angle (φ and Ψ) was retained for each residue in the protein and transformed into a RDP by using Kernel density estimation with a grid dimension of 32 × 32. The "training data" was calculated by averaging each grid point for wildtype (WT) and benign variants. Pathogenic variants followed the same procedure, and each grid point of the respective RDP was compared with the training data. The grid point was marked as significant structural deviation if it was beyond the standard deviation of the training data.

The optimal cut-off points for determining benign and pathogenic variants were determined by shifting the cut-off point with a bin size of 0.01 in the range of 2.7–4.0. Each variant prediction was assigned as TP if the model predicted the variant as deleterious and the database classified as pathogenic, or as TN if the model predicted as "unknown" and the database classified as benign, or as FP if the model predicted as deleterious but the database classified as benign, or FN if the model predicted as "unknown" but the database classified as pathogenic. The sum of TP, TN, FP, and FN variants was documented for each bin. The overlapped percentage of TP and TN were assumed to be the optimal cut-off point to classify the variants as "deleterious" or "unknown".

#### Machine learning

The process of the DL-RP-MDS is presented in Figure 1. The initial process and system setup followed our previous publication.^6^ Here, we included DL in the analysis stage for examining individual RSP. The following sections showed the details of AE and the multi-label classifier.

#### Nonlinear dimensionality reduction: Auto-encoder (AE)

An AE is a feedforward, non-recurrent neural network, which consists of the encoder and the decoder.^11^^,^^12^ $E:R^{p}\to R^{q}$ is an operator for the encoder and $D:R^{q}\to R^{p}$ for the decoder, each with *M* hidden layers, where *p* is the input/output dimension and *q* is the latent dimension. The encoder stage of the AE takes the input $X\in R^{p}$ and maps it to the latent representation $H\in R^{q}$:

(Equation 1) $$H=E\left(X\right)=W_{M+1}\circ \left(\sigma_{M}\circ W_{M}\right)\circ \cdots \circ \left(\sigma_{1}\circ W_{1}\right)\left(Χ\right)$$

where $W_{M}$ is the weight matrix including the bias vector between the *m*^*th*^ and the (*m* + 1)^th^ layers, $\sigma_{m}:R\to R$ is the activation function for each 'node' in the *m*^th^ layer, and ◦ denotes operator composition. The decoder stage of the AE maps the latent representation H back to the reconstructed $X^{\prime }\in R^{p}$:

(Equation 2) $$X^{\prime }=D\left(H\right)=W_{M+1}^{\prime }\circ \left(\sigma_{M}^{\prime }\circ W_{M}^{\prime }\right)\circ \cdots \circ \left(\sigma_{1}^{\prime }\circ W_{1}^{\prime }\right)\left(H\right)$$

where we decided to use $\sigma_{m}^{\prime }=\sigma_{m}$ when building the AE.

We used a time-one-lagged AE to find low-dimensional representations of the MD simulations, which is shown to outperform those found by other 'conventional' methods, such as PCA.^45^ We refer to it as an AE in this study. Figure 2 shows an example of the latent representation for MD simulations. The AE was trained to minimize a reconstruction error. We used the mean squared error as the loss function:

(Equation 3) $$L\left(X,X^{*}\right)=\frac{1}{N}{\left\|X^{*}-D\left(\varepsilon \left(X\right)\right)\right\|}_{2}^{2}$$

where X is the input (standardized) atomic configuration (φ and Ψ angles) at time *t* to be encoded, ***X∗*** is the torsional configuration of the trained data at time *t* + 1,^45^ ${\left\|\cdot \right\|}_{2}$ denotes the $L^{2}$ norm and *N* input data points. The weights and biases were initialized randomly using the Glorot uniform initialization method, and the optimization was performed using Adam’s algorithm with a learning rate of 0.001.^69^ For the AE architecture and design, we followed the procedure in ref.^45^; we used the Leaky-Rectified Linear Unit (ReLU) as the activation function *σ* and implements two hidden layers (*M* = 2) with 1,000 nodes between the input and latent layers and dropout rate of 0.1.

#### Multi-label classification: Multi-layer neural network classifier

After encoding the complex torsional configurations from the MD simulations into the latent representation using the time-lagged AE, we performed classification for the data as deleterious or undefined function. We employed a fully connected neural network model $N:R^{q}\to {\left[0,1\right]}^{2}$ with $M^{\prime \prime }$ hidden layers as the multi-label classification model, which can be expressed as

(Equation 4) $$L=N\left(H\right)=\left(\sigma_{M^{\prime \prime }+1}^{\prime \prime }\circ W_{M^{\prime \prime }+1}^{\prime \prime }\right)\circ \left(\sigma_{M^{\prime \prime }}^{\prime \prime }\circ W_{M^{\prime \prime }}^{\prime \prime }\right)\circ \cdots \circ \left(\sigma_{1}^{\prime \prime }\circ W_{1}^{\prime \prime }\right)\left(H\right)$$

where the model took the standardized latent representation from the AE as the inputs and mapped the inputs to two labels representing the probability of the variant being a deleterious or undefined function. We used the Leaky-ReLU as the activation function for the hidden layers (*σ*_*m*_ for *m* ≤ *“M”*) and the sigmoid activation function for the output (*σ*_*M’’+1*_). As the data for the classification were imbalanced due to the smaller number of Benign variants than that of pathogenic variants, SMOTE was employed to enhance Benign variants data.^15^ The neural network classifier was trained as described for the AE. We used the cross-entropy as the loss function:

(Equation 5) $$L^{\prime \prime }\left(H,L^{*}\right)=-\sum_{i=1}^{2}L_{i}^{*}\;\log\left(N{\left(H\right)}_{i}\right)$$

where $L^{*}$ is ground-truth labels. The weights and biases were initialized randomly using the Glorot uniform initialization method, and the optimization was performed using Adam’s algorithm with a learning rate of 0.001. Bayesian optimizations were performed for hyperparameter tuning for the neural network classifier to determine the best architecture, combined with a grid search for the number of latent dimensions.

### Quantification and statistical analysis

#### F_2_ score validation and accuracy test

We use the *F*_2_ score of the validation dataset as the objective function of the hyperparameter tuning:

(Equation 6) $$F_{2}=\left(1+\beta^{2}\right)\frac{\text{precision}\times \text{recall}}{\beta^{2}\times \text{precision}+\text{recall}}$$

where precision=TP/(TP+FP) and recall=TP/(TP+FN) and *β* = 2. The computed accuracy of the predictions for comparison was calculated by

(Equation 7) $$\text{accuracy}=\frac{TP+TN}{TP+FP+TN+FN}$$

To obtain the final probability of a variant as a deleterious or undefined function, we took the mean value of the classification probability using the full-time series of the MD simulation results. The final classification of the variant was based on the highest probability of the prediction. For example, if P(variantisdeleterious,D)=0.7 and P(variantisundefined,U)=0.3, the variant was labelled as deleterious, and vice versa.

#### Statistical analysis and cross-validation test

A four-fold stratified cross-validation test with five repeats was performed to assess the sensitivity and specificity of RP-MDS and DL-RP-MDS for all datasets.^70^ In brief, the benign and pathogenic Ramachandran plots were randomly permuted and divided into four groups. In each run, one of the groups was selected as the testing dataset and the remained groups were used as the training dataset for model training. A total of 20 models were created for RP-MDS and DL-RP-MDS, and the performances were represented by the ROC curve and AUC. The ROC curve for RP-MDS was generated by shifting the cut-off with an equal bin size of 0.01, and the ROC curve for DL-RP-MDS was calculated by using python library Scikit-learn.

Further, we used the balanced accuracy (BA) to calculate the average true negative rate (TNR) and true positive rate (TPR) of the variants classified by the model:

(Equation 8) $$\text{balance}\;\text{accuracy}=\frac{TPR+TNR}{2}$$

The TPR and TNR values for RP-MDS were calculated based on the optimal cut-off value, and TPR and TNR values for DL-RP-MDS were calculated based on the results generated from the model.

## Data Availability

• The DL-RP-MDS code is available in: https://doi.org/10.5281/zenodo.7435215. • The DL-RP-MDS website is available in: https://genemutation.fhs.um.edu.mo/DL-RP-MDS/. • Any additional information required to reanalyze the data reported in this paper is available from the lead contact upon request.

## Supplemental information

Document S1. Figures S1–S4

Table S1. Summary of the known variants used in the study (ClinVar) (A) Known TP53 variants used in the study (ClinVar). (B) Known MLH1 variants used in this study (ClinVar). (C) Known MSH2 variants used in the study (ClinVar).

Table S2. Classification results of the variants by RP-MDS and DL-RP-MDS (A) Classification of TP53 variants by RP-MDS and DL-RP-MDS, related to Figure 5 and Table 2. (B) Classification of MLH1 variants by RP-MDS and DL-RP-MDS, related to Figure 5 and Table 2. (C) Classification of MSH2 variants by RP-MDS and DL-RP-MDS, related to Figure 5 and Table 2.

Table S3. Comparison of DL-RP-MDS with other in silico methods for classification of the variants (A) Comparison of DL-RP-MDS with other in silico methods for classification of TP53 variants, related to Table 2. (B) Comparison of DL-RP-MDS with other in silico methods for classification of MLH1 variants, related to Table 2. (C) Comparison of DL-RP-MDS with other in silico methods for classification of MSH2 variants, related to Table 2.
