# Supplementary material for: Integration of deep learning with Ramachandran plot molecular dynamics simulation for genetic variant classification
Source: iScience. 2023 Feb 2;26(3):106122. doi: 10.1016/j.isci.2023.106122 (PMC9984559; doi:10.1016/j.isci.2023.106122)
Supplement: Document S1. Figures S1–S4 [file mmc1.pdf]

iScience, Volume *26*

Supplemental information

Integration of deep learning with Ramachandran plot molecular dynamics simulation for genetic variant classification

Benjamin Tam, Zixin Qin, Bojin Zhao, San Ming Wang, and Chon Lok Lei

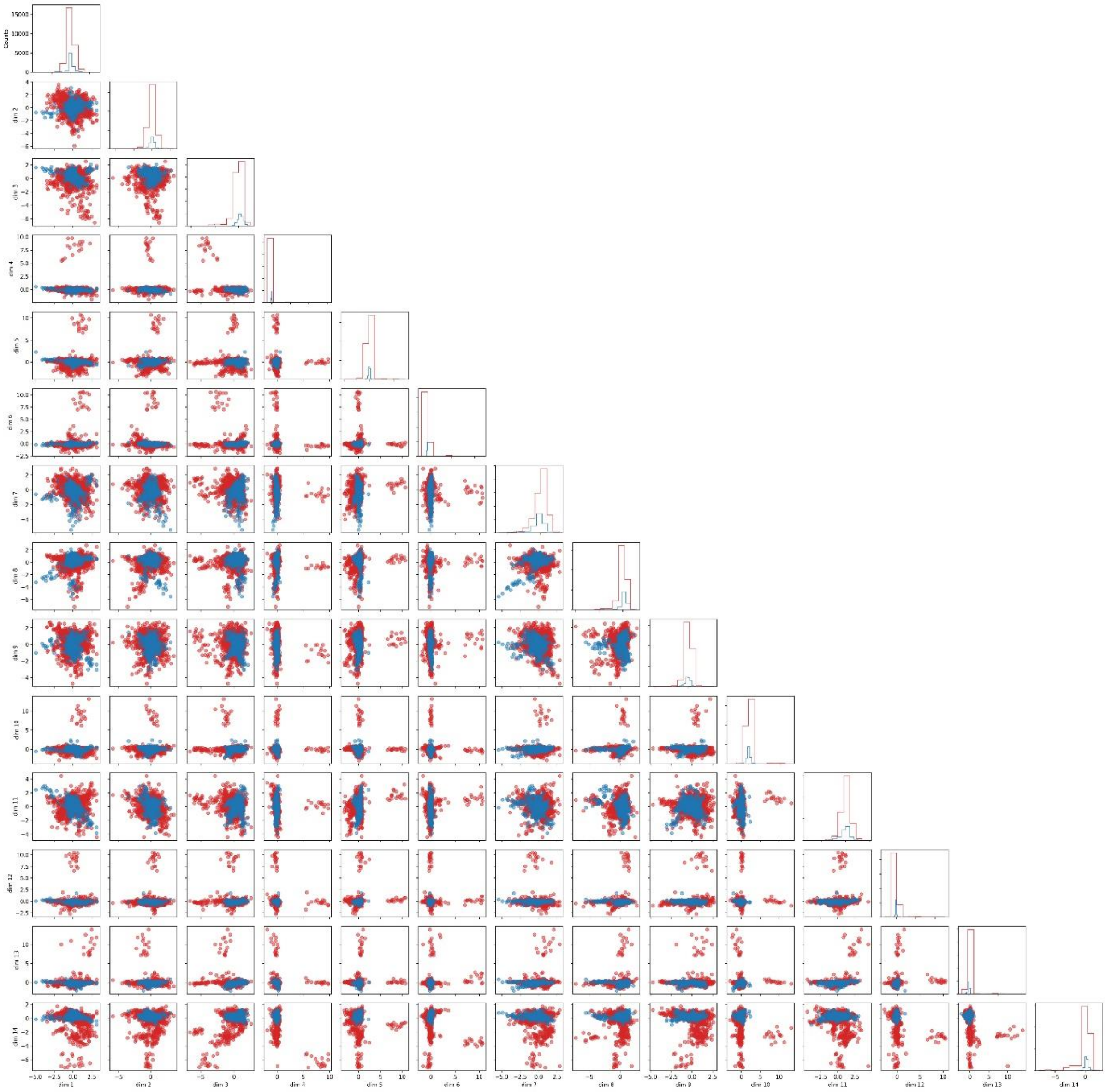

Figure S1 – Latent dimensions of TP53. Related to figure 3. DL-RP-MDS reduced the complexity of Ramachandran scatter plots but retained the crucial information. The combination of the unique information in each dimension was used as the classification criteria for TP53. Blue: Benign variants; Red: Pathogenic variants.

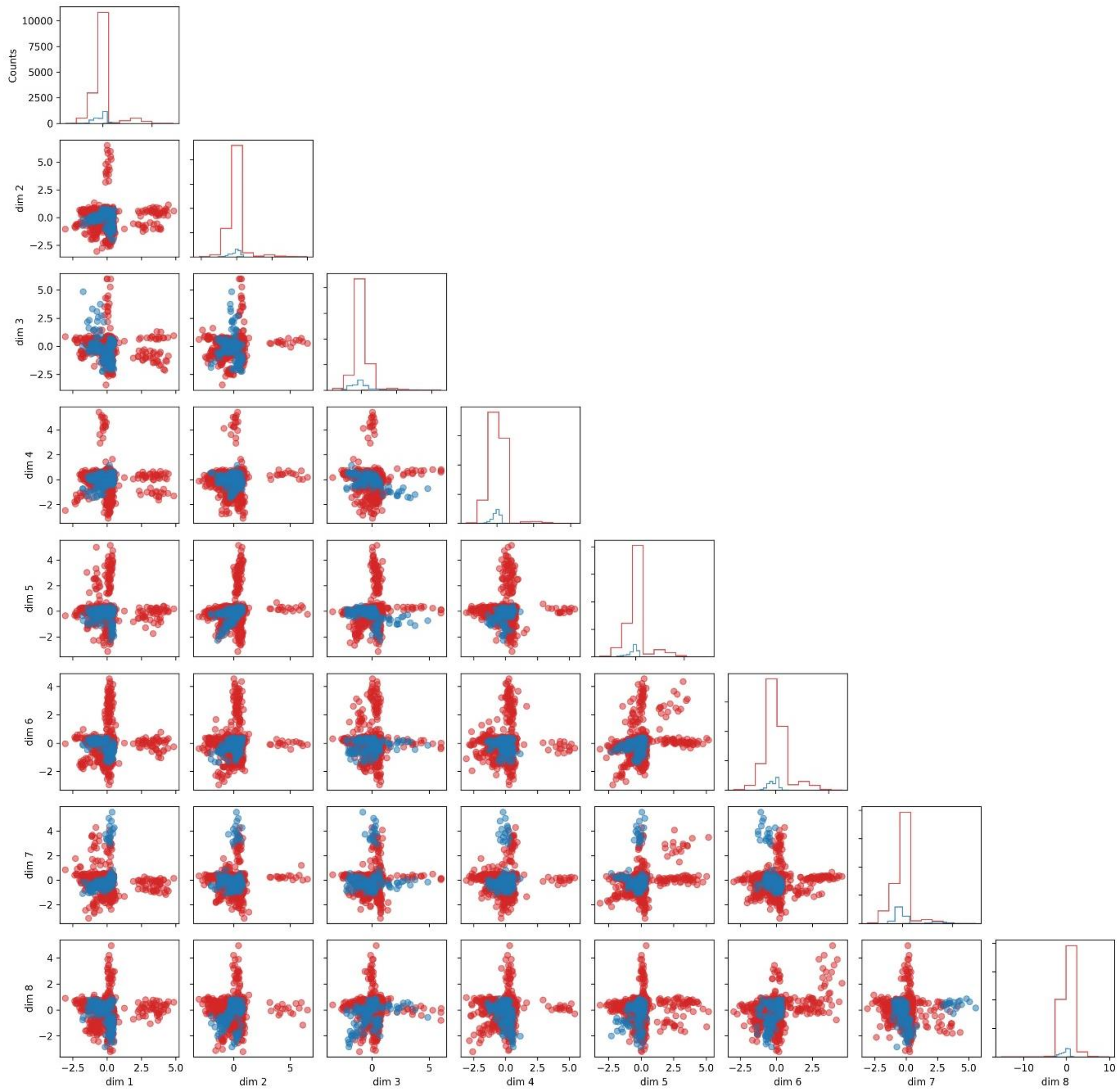

Figure S2 – Latent dimensions of MLH1. Related to figure 3. DL-RP-MDS reduced the complexity of Ramachandran scatter plots but retained the crucial information. The combination of the unique information in each dimension was used as the classification criteria for MLH1. Blue: Benign variants; Red: Pathogenic variants.

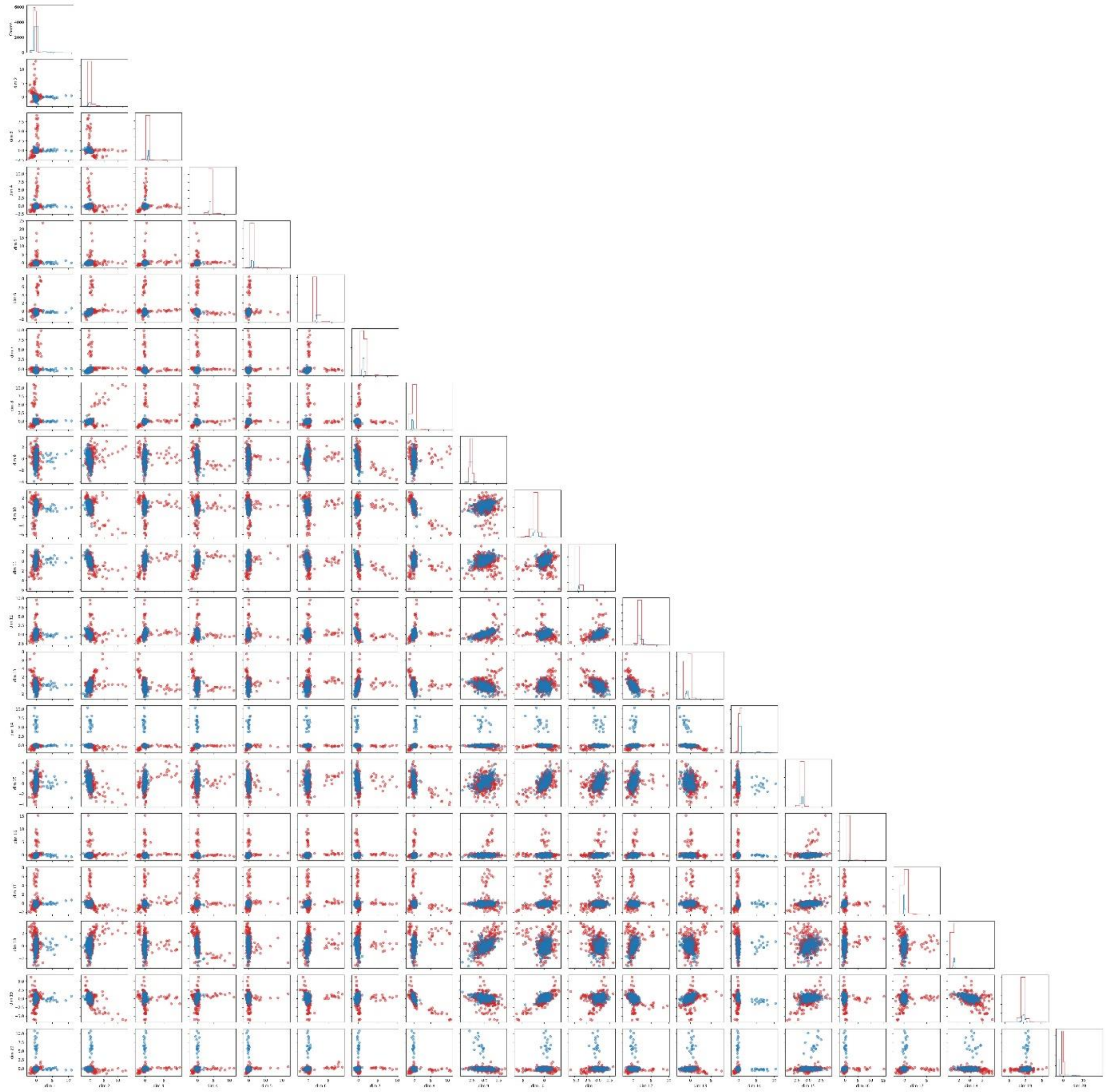

Figure S3 – Latent dimensions of MSH2. Related to figure 3. DL-RP-MDS reduced the complexity of Ramachandran scatter plots but retained the crucial information. The combination of the unique information in each dimension was used as the classification criteria for MSH2. Blue: Benign variants; Red: Pathogenic variants.

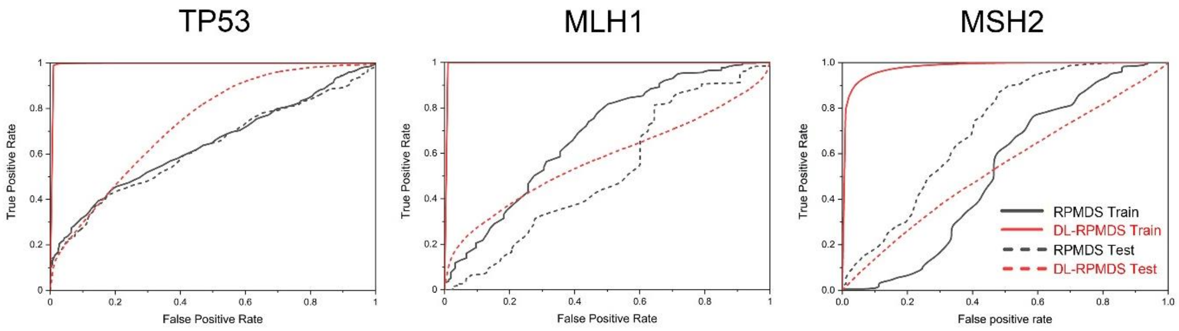

Figure S4 – Receiver operating characteristic (ROC) curves for DL-RP-MDS and RP-MDS. Related to table 1 and figure 4. Each ROC curve was the average of 20 stratified cross-validation models. For DL-RP-MDS, variants were grouped by variants. The testing dataset is lower than the training dataset as illustrated by the under the curve (AUC) for TP53 (left), MLH1 (center), and MSH2 (right). For RP-MDS, the AUC of the testing dataset and training dataset was comparable for TP53, lower for MLH1 and higher for MSH2. Red solid line: DL-RP-MDS training dataset model; Red dotted line: DL-RP-MDS testing dataset model; Black solid line: RP-MDS training dataset model; Black dotted line: RP-MDS testing dataset model.
